# Adrenal oncocytoma: a rare presentation of a benign ^18^F-fluorodeoxyglucose PET avid virilising adrenal tumour

**DOI:** 10.1097/MNM.0000000000001932

**Published:** 2024-11-28

**Authors:** Danielle Lee, Jonathan Green, James Crane, David R. Taylor, Saira Reynolds, Wen Ng, Koshy Jacob, Benjamin Whitelaw, Simon Aylwin, Gabriele Galata, Dylan Lewis, Matthew Seager

**Affiliations:** a King’s College Hospital NHS Foundation Trust,; b Faculty of Life Sciences and Medicine, King’s College London; c Department of Clinical Biochemistry (Synnovis), King’s College Hospital NHS Foundation Trust, London and; d Eastbourne District General Hospital, Eastbourne, UK

**Keywords:** adrenal gland neoplasms, adrenocortical oncocytoma, F-18 fluorodeoxyglucose-PET, hyperandrogenism

## Abstract

A woman in her 70s presented with features of hyperandrogenism including clitoral enlargement and deepening of her voice. Biochemical investigations revealed raised plasma androgens and urinary androgen metabolites and imaging findings showed a highly F-18 fluorodeoxyglucose (FDG)-PET avid left adrenal tumour initially suspected to be a malignant adrenocortical carcinoma (ACC). She subsequently underwent an uncomplicated laparoscopic adrenalectomy where complete resection of her tumour was achieved. Histopathological analysis demonstrated a benign adrenal oncocytoma with no evidence of malignancy. This case illustrates a rare presentation of a functioning virilising adrenal oncocytoma as a benign mimic of ACC.

## Case presentation

**Fig. 1:**
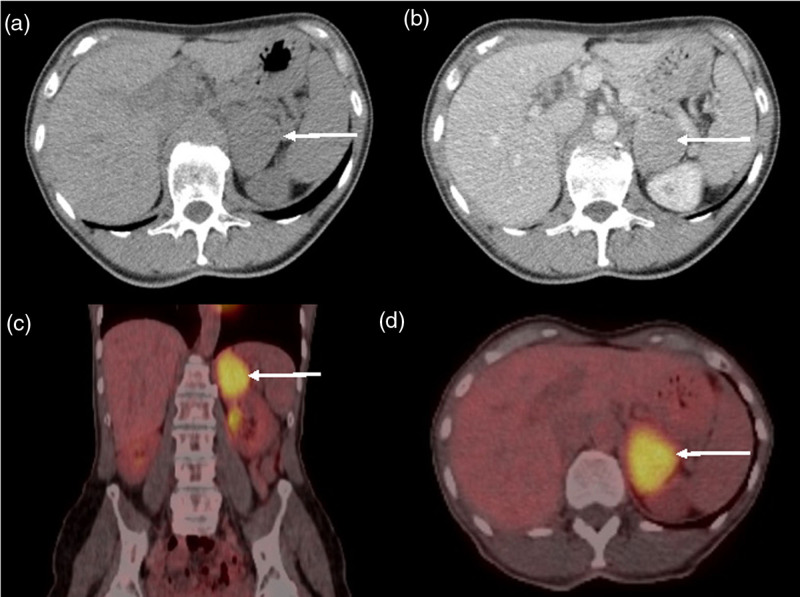
Preoperative computed tomography (CT) scans. Noncontrast CT (a) of left adrenal mass (white arrow) shows a mean attenuation of 43 HU with enhancement on the portal venous phase (b) with mean attenuation of 88 HU. Coronal (c) and axial (d) F-18 fluorodeoxyglucose (FDG) PET findings show significant tracer uptake in the left adrenal mass, with a maximum standardised uptake value (SUVmax) of 15, and adrenal tumour to liver SUVmax ratio of 5.

A woman in her 70s presented to her general practitioner after noticing that her voice had dropped in vocal range from soprano to alto as a singer with her local community choir. She had also noted clitoral enlargement over the preceding year. She was otherwise well with no past medical history other than gastro-oesophageal reflux. On examination, she was noted to have clitoromegaly but no other features of virilisation. She was referred to her local endocrinology team for further assessment, and a computed tomography (CT) scan was arranged, which identified a 6 cm left adrenal mass (Fig. 1) for which she was referred to our tertiary endocrinology centre for discussion in our adrenal multi-disciplinary team meeting.

## Investigations

Her blood tests showed raised testosterone 3.5 nmol/L (normal 0.4–1.4 nmol/L) and dehydroepiandrosterone SO4 (DHEAS) 14.8 μmol/L (normal 0.9–11.6 μmol/L). Her adrenal biochemistry was otherwise normal, including normal plasma metanephrines, urinary cortisol, overnight dexamethasone suppression test, renin and aldosterone.

Her urine steroid profile showed high levels of the major androstenedione/testosterone metabolites androsterone 1936 μg/24 h (normal adult female mean 770 μg/24 h, SD 420 μg/24 h) and aetiocholanolone 4672 μg/24 h (normal adult female mean 937 μg/24 h, SD 621 μg/24 h), with an associated increase in dehydroandrosterone (DHA) 852 μg/24 h (normal adult female mean 327 μg/24 h, SD 244 μg/24 h) and its metabolites 16αOH-DHA 2229 μg/24 h (normal adult female mean 205 μg/24 h, SD 231 μg/24 h) and androstenetriol 2016 μg/24 h (normal adult female mean 294 μg/24 h, SD 234 μg/24 h). There were otherwise no increases in other abnormal steroid metabolites that would typically be associated with adrenocortical carcinoma (ACC) [1].

**Fig. 2:**
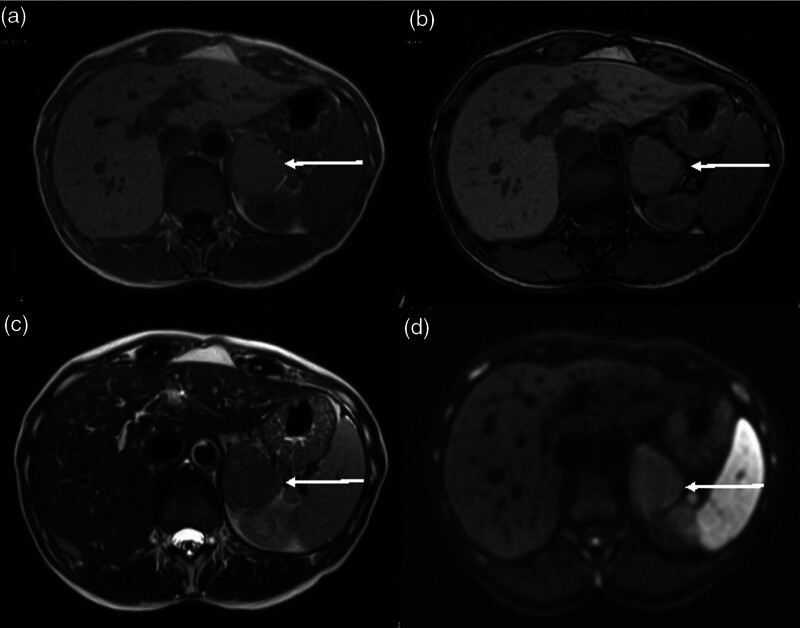
Preoperative axial MRI imaging. In phase T1 (a) and opposed phase T1 (b) images show no evidence of intravoxel fat within the mass. T2 (c) imaging showing intermediate signal intensity. DWI B100 image (d) showing mild increased signal in the mass with corresponding reduced signal on the ADC map (not shown), indicating mild restricted diffusion. ADC, apparent diffusion coefficient; DWI, diffusion-weighted imaging

Her adrenal CT and F-18 fluorodeoxyglucose PET (FDG-PET) scans showed an enhancing and highly FDG-avid left adrenal mass and no evidence of metastatic disease (Fig. 1). Her adrenal MRI showed intermediate T2 signal intensity, no intravoxel fat and mild restricted diffusion (Fig. 2).

## Differential diagnosis

Her clinical presentation of hyperandrogenism, biochemical findings of raised plasma androgens and urinary androgen metabolites, and radiological findings of a highly FDG-PET avid adrenal lesion were initially felt to be suspicious for ACC.

ACC is a rare adrenal cortex malignancy with an estimated incidence of 1–2 per million a year. It is usually associated with a poor prognosis with a 5-year survival of <15% in metastatic disease [2]. Up to two-thirds of ACC cases present with clinical and/or biochemical features of steroid hormone excess, with adrenal androgens being the second most commonly produced hormones after cortisol in hormone-secreting ACCs [3]. The gold standard for diagnosis is via histopathological analysis, with surgical resection being recommended over biopsy as a means to obtaining a tissue diagnosis in order to minimise the risk of tumour dissemination [2].

## Treatment

The decision was therefore made for her to undergo surgical resection of her tumour in view of the high suspicion for ACC. She underwent an uncomplicated laparoscopic adrenalectomy, during which complete resection of her adrenal tumour was achieved. Intraoperatively, she was noted to have no evidence of organ or vascular invasion, with no metastatic lymph nodes.

## Histopathological diagnosis

**Fig. 3:**
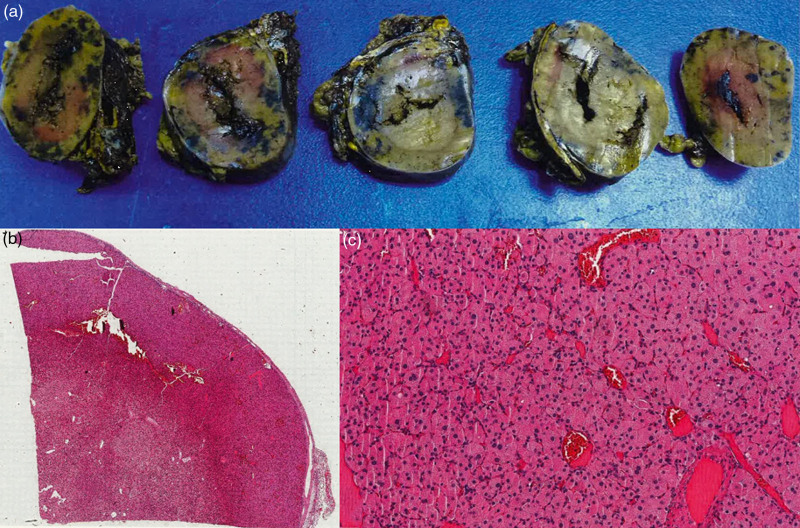
Left adrenal gland histopathological analysis showing a 58 mm adrenal mass with an intact capsule and complete excision macroscopically (a). Microscopic analysis (b, c) showed positive staining for inhibin, calretinin and MelanA with a low proliferation Ki67 index of 1–2.2%.

Histopathological analysis of her adrenal tumour demonstrated this to be an oncocytic adrenocortical adenoma with a low proliferation index (Ki67: 1–2.2%) and staining positively for inhibin, calretinin and MelanA. Macroscopically, there was evidence of complete tumour excision with an intact capsule and no histological evidence of metastasis (Fig. 3). Multiple risk scoring systems namely the reticulin algorithm, Lin-Weiss-Biscelgia (LWB), and Helsinki system, were used to assess for malignancy, all of which were negative.

The Weiss system is the most widely used system for determining the malignant potential of adrenocortical tumours in adults and assesses for the presence of nine morphological criteria related to tumour structure, cytological features and evidence of tumour invasion, with the presence of three or more features being consistent with a diagnosis of ACC [4]. However, its use in oncocytic neoplasms is more limited, due to the features of eosinophilic cytoplasm, diffuse architecture and high nuclear grade being present universally in these tumours regardless of malignant potential, therefore resulting in a falsely high Weiss score and subsequently a false diagnosis of malignancy [5].

The updated 2022 WHO classification of adrenal cortical tumours, therefore, recommends the use of alternative scoring systems in the assessment of the malignant potential of oncocytic adrenal neoplasms, namely the LWB system, the Helsinki score, and the reticulin algorithm [6]. The LWB system was first developed in 2004 specifically for assessment of oncocytic variant adrenal neoplasms and defines the diagnosis of ACC by the presence of at least one ‘major criteria’ (mitosis >5, atypical mitosis or vascular invasion) [7]. However, some studies have shown that this system can have the potential to overestimate malignant potential of oncocytic adrenocortical tumours [8].

The reticulin algorithm incorporates the presence of an altered reticulin network on a Gordon Sweet silver histochemical stain alongside one of the following parameters of high mitotic rate, tumour necrosis and vascular invasion to determine a diagnosis of ACC [9]. This method has been shown to have good interobserver reproducibility for the diagnosis of ACC [10], and to accurately identify malignant oncocytic adrenocortical tumours which follow an aggressive course [11].

The Helsinki score is viewed as the most reliable in differentiating between malignant and benign adrenocortical neoplasms, with a sensitivity of 100% and specificity of 99.6% [12]. Studies have also shown that it has the highest specificity for the identification of malignant oncocytic adrenocortical tumours compared with other scoring systems [8]. It is the only scoring system that integrates the Ki67 proliferation index into its overall score, alongside mitotic rate and evidence of tumour necrosis, with a score of >8.5 being diagnostic for ACC, and a score of >17 being associated with a poor prognosis [13]. However, it does have the potential to underestimate the malignant potential of oncocytic tumours, due to the increased difficulty with assessing mitoses in these cells [5].

In this case, histopathological analysis of the tumour demonstrated an LWB score of 0, a reticulin algorithm score of 0 and a Helsinki score of 2.2, which was consistent with a benign adrenal oncocytoma across all scoring systems.

## Treatment outcome

**Fig. 4:**
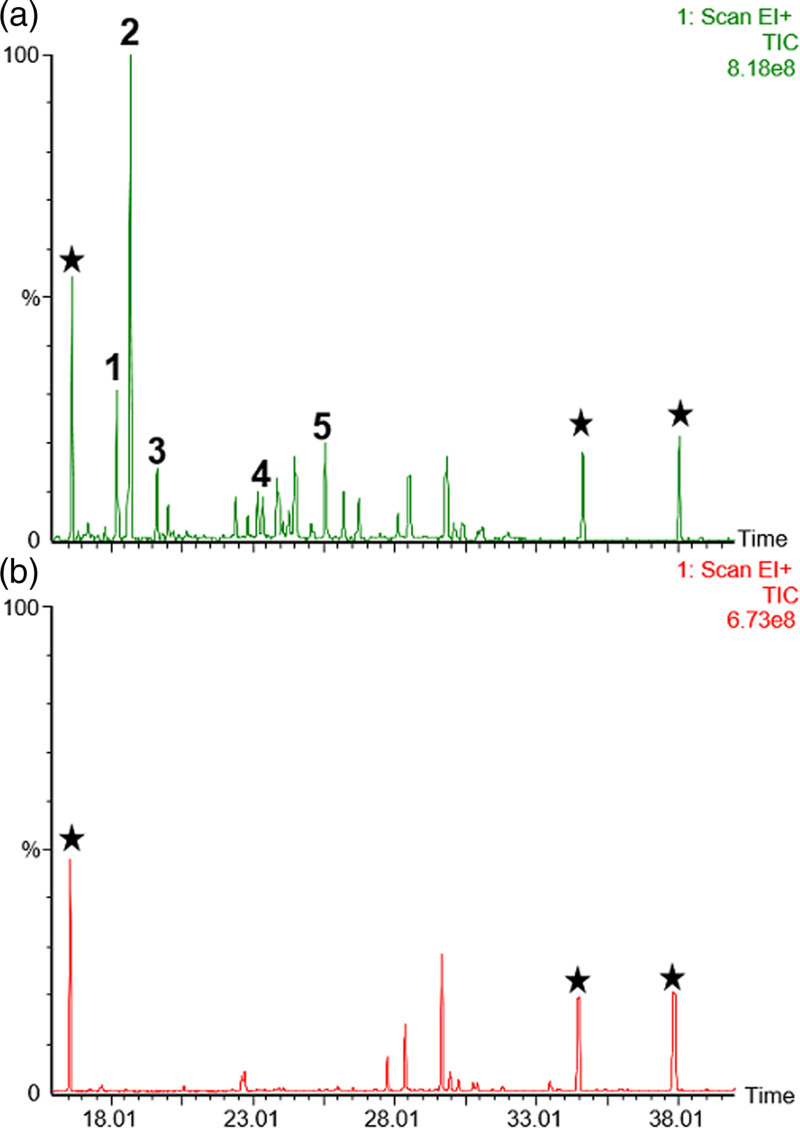
Total ion current chromatograms from gas chromatography-mass spectrometry (GC–MS) analysis of urinary steroid metabolites excreted (a) by the patient at diagnosis, (b) after her adrenalectomy. The abnormal steroid metabolites labelled in the preoperative sample are (1) androsterone, (2) aetiocholanolone (both derived from androstenedione and testosterone), (3) DHA, (4) 16αOH-DHA and (5) androstenetriol (all derived from DHA). Postoperatively these steroid metabolites were no longer increased. The black stars indicate the position of exogenous internal standards.

She has made a full recovery following her surgery and currently remains under regular endocrinology outpatient follow-up with biochemical surveillance of her testosterone and DHEAS. Her postoperative short-synacthen test showed evidence of ongoing adrenal insufficiency for which she remains on hydrocortisone, with a basal cortisol of 81 nmol/L, rising to only 120 nmol/L after 30 min. She has otherwise remained in biochemical remission 1-year following her surgery, with her latest biochemistry showing normalisation of the urine steroid profile (Fig. 4) and serum androgens [DHES < 0.4 μmol/L (normal: 0.9–11.6 μmol/L) and testosterone <0.4 nmol/L (normal: 0.4–1.4 nmol/L)]. She, however, has not yet noticed any significant improvements to her symptoms of deepening voice or clitoromegaly 1-year following her surgery.

## Discussion

Adrenal oncocytomas are rare tumours, which are predominantly benign and hormonally nonfunctional. Around 287 cases of adrenal oncocytic neoplasm have been described in the literature, with around 30% being described as functional and 30% being found to be malignant [14]. Oncocytomas are tumours where the dominant cell type consists of cells containing granular cytoplasmic eosinophilia due to numerous mitochondria [15]. They originate from epithelial cells and occur mainly in endocrine and exocrine organs such as the thyroid, parathyroids, kidneys, salivary gland and pituitary gland [16].

Imaging findings in adrenal oncocytoma are variable, although are typically characterised as well-demarcated solid encapsulated masses on CT imaging [17]. Postcontrast, these lesions tend to demonstrate heterogeneous enhancement, and in contrast to renal oncocytoma they usually lack a central scar [18]. On MRI, typical findings of adrenal oncocytoma are a heterogeneous appearance in T1 and T2 weighted sequences, with hyperintensity of T2 and variable isointense to hypointense signal on T1. Diffusion weighted imaging findings are also variable, with these lesions usually showing moderate to intense diffusion restriction. They tend to show moderate enhancement following administration of gadolinium [19].

Benign lesions do not typically demonstrate high PET-avidity. However, several reports of highly PET-avid benign adrenal oncocytoma have been reported in the literature [20–25]. The mechanism of high FDG uptake in adrenal oncocytoma is not completely understood but is thought to be due to the presence of large numbers of intracellular mitochondria and resulting increased glucose utilisation [21,26].

FDG-PET has overall been shown to have good sensitivity and specificity for characterisation of adrenal masses, with a recent meta-analysis showing an overall pooled sensitivity of 87.3% and specificity of 84.7% for the use of FDG-PET in the differentiation between benign and malignant adrenal lesions [27]. Analysis can take the form of either qualitative assessment by visual evaluation, or quantitative assessment by using adrenal SUVs (standardised uptake values) or the adrenal lesion-to-liver SUV ratio. Studies have reported optimal adrenal SUVmax cut-off values for malignancy ranging from 2.1 and 4.1, and adrenal lesion-to-liver SUV ratio cut-off values ranging from 1.08 to 2.5 [28]. These have been shown to have overall high sensitivity (86–100% for SUVmax, and 85–100% for SUVratio), but lower specificity (66–75% for SUVmax, and 70–100% for SUVratio) for the detection of malignancy [28].

However, studies have demonstrated that benign adrenal lesions can exhibit variable FDG-PET avidity, resulting in false-positive diagnoses for malignancy [26]. Our case illustrates a further example of this, with her FDG-PET scan demonstrating an adrenal lesion SUVmax of 15, and an adrenal lesion-to-liver SUVmax ratio of 5, both of which fall within what is conventionally regarded a malignant range. There is also evidence that some functional adrenal lesions, such as phaeochromocytoma, show increased FDG uptake compared with nonfunctional lesions, although the uptake intensity is variable between those which are malignant and benign, therefore limiting the utility of FDG-PET in differentiating benign from malignant lesions in these cases [29]. Other benign lesions that have demonstrated increased 18F-FDG-PET uptake include adrenal adenoma and hyperplasia, as well as infectious/inflammatory processes [30].

### Conclusion

Adrenal oncocytoma should be considered a rare benign differential diagnosis of an FDG-PET avid adrenal lesion. Histopathological analysis is essential in the diagnosis of adrenal oncocytoma, and surgical resection is, therefore, the mainstay of management, both for the purposes of confirming a histological diagnosis, and as a curative treatment.

## Acknowledgements

Written consent was obtained from the patient to publish this article.

### Conflicts of interest

There are no conflicts of interest.

## References

[1] Arlt W Biehl M Taylor AE Hahner S Libé R Hughes BA. Urine steroid metabolomics as a biomarker tool for detecting malignancy in adrenal tumors. J Clin Endocrinol Metab 2011; 96:3775–3784. 21917861 10.1210/jc.2011-1565 PMC3232629

[2] Libé R Huillard O. Adrenocortical carcinoma: diagnosis, prognostic classification and treatment of localized and advanced disease. Cancer Treat Res Commun 2023; 37:100759. 37690343 10.1016/j.ctarc.2023.100759

[3] Lam AKY. Adrenocortical carcinoma: updates of clinical and pathological features after renewed world health organisation classification and pathology staging. Biomedicines 2021; 9:1–25. 10.3390/biomedicines9020175 PMC7916702 33578929

[4] Papotti M Libè R Duregon E Volante M Bertherat J Tissier F. The Weiss score and beyond – histopathology for adrenocortical carcinoma. Horm Cancer 2011; 2:333–340. 21997290 10.1007/s12672-011-0088-0 PMC10358021

[5] Urusova L Porubayeva E Pachuashvili N Elfimova A Beltsevich D Mokrysheva N. The new histological system for the diagnosis of adrenocortical cancer. Front Endocrinol (Lausanne) 2023; 14:1218686. 37560295 10.3389/fendo.2023.1218686 PMC10406575

[6] Mete O Erickson LA Juhlin CC de Krijger RR Sasano H Volante M Papotti MG. Overview of the 2022 WHO classification of adrenal cortical tumors. Endocr Pathol 2022; 33:155–196. 35288842 10.1007/s12022-022-09710-8 PMC8920443

[7] Bisceglia M Ludovico O Di Mattia A Ben-Dor D Sandbank J Pasquinelli G. Adrenocortical oncocytic tumors: report of 10 cases and review of the literature. Int J Surg Pathol 2004; 12:231–243. 15306935 10.1177/106689690401200304

[8] Renaudin K Smati S Wargny M Al Ghuzlan A Aubert S Leteurtre E; for Comete-Cancer Network. Clinicopathological description of 43 oncocytic adrenocortical tumors: importance of Ki-67 in histoprognostic evaluation. Mod Pathol 2018; 31:1708–1716. 29921900 10.1038/s41379-018-0077-8

[9] Volante M Bollito E Sperone P Tavaglione V Daffara F Porpiglia F. Clinicopathological study of a series of 92 adrenocortical carcinomas: from a proposal of simplified diagnostic algorithm to prognostic stratification. Histopathology 2016; 55:535–543. 10.1111/j.1365-2559.2009.03423.x 19912359

[10] Duregon E Fassina A Volante M Nesi G Santi R Gatti G. The reticulin algorithm for adrenocortical tumor diagnosis: a multicentric validation study on 245 unpublished cases. Am J Surg Pathol 2016; 37:1433–1440. 10.1097/PAS.0b013e31828d387b 23774167

[11] Duregon E Volante M Cappia S Cuccurullo A Bisceglia M Wong DD. Oncocytic adrenocortical tumors: diagnostic algorithm and mitochondrial DNA profile in 27 cases. Am J Surg Pathol 2023; 35:1882–1893. 10.1097/PAS.0b013e31822da401 21989346

[12] Minner S Schreiner J Saeger W. Adrenal cancer: relevance of different grading systems and subtypes. *Clin Transl Oncol* 2021; 23:1350–1357. 10.1007/s12094-020-02524-2 PMC8192347 33818702

[13] Pennanen M Heiskanen I Sane T Remes S Mustonen H Haglund C Arola J. Helsinki score – a novel model for prediction of metastases in adrenocortical carcinomas. Hum Pathol 2015; 46:404–410. 25582500 10.1016/j.humpath.2014.11.015

[14] Coppola Bottazzi E Gambardella C Mongardini FM Vanella S Noviello A Palma T. Prognosis of adrenal oncocytic neoplasms (AONs): literature review of 287 cases and presentation of the oldest patient. J Clin Med 2023; 12:6925. 37959390 10.3390/jcm12216925 PMC10649738

[15] Cotton DWK. Oncocytomas. Histopathology 1990; 16:507–509. 2193866 10.1111/j.1365-2559.1990.tb01555.x

[16] Gasparre G Romeo G Rugolo M Porcelli AM. Learning from oncocytic tumors: why choose inefficient mitochondria? Biochim Biophys Acta 2011; 1807:633–642. 20732299 10.1016/j.bbabio.2010.08.006

[17] Muñoz de Nova JL García-Sanz I del Campo Val L Delgado Valdueza J Martín-Pérez E. Oncocytoma: an uncommon lesion for adrenal gland. Endocrinol Nutr. 2015; 62:144–145. 25648702 10.1016/j.endonu.2014.12.006

[18] Coppola M Romeo V Verde F Raia G Mainolfi CG Aprea G. Integrated imaging of adrenal oncocytoma: a case of diagnostic challenge. Quant Imaging Med Surg. 2019; 9:1896–1901. 31867239 10.21037/qims.2019.06.20 PMC6902136

[19] Giesteira B Sousa J Amorim JP. Incidental adrenal masses: a case report of an adrenal oncocytoma. Cureus 2023; 15:e47994. 38034194 10.7759/cureus.47994 PMC10686665

[20] Kim DJ Chung JJ Ryu YH Hong SW Yu J-S Kim JH. Adrenocortical oncocytoma displaying intense activity on 18F-FDG-PET: a case report and a literature review. Ann Nucl Med 2008; 22:821–824. 19039562 10.1007/s12149-008-0199-z

[21] Kakita-Kobayashi M Usui T Sasano H Shimatsu A. 18F-FDG-PET-positive adrenal tumour. Case Rep 2015; 2015:bcr2015209379. 10.1136/bcr-2015-209379 PMC4368916 25750227

[22] Son SH Lee SW Song B-I Jang Y-J Park J-Y Jeong SY. Recurrence of a functional adrenocortical oncocytoma of borderline malignant potential showing high FDG uptake on 18F-FDG PET/CT. Ann Nucl Med 2014; 28:69–73. 23990396 10.1007/s12149-013-0764-y

[23] Sato N Nakamura Y Takanami K Ono Y Omata K Morimoto R. Case report: adrenal oncocytoma associated with markedly increased FDG uptake and immunohistochemically positive for GLUT1. Endocr Pathol 2014; 25:410–415. 25284122 10.1007/s12022-014-9337-4

[24] Canu L Perigli G Badii B Santi R Nesi G Pradella S. Case report: adrenocortical oncocytoma in a patient with a previous contralateral adrenalectomy for a cortisol-secreting adenoma. Front Surg 2022; 9:897967. 35662823 10.3389/fsurg.2022.897967 PMC9160572

[25] Acar C Akkas BE Sen I Sozen S Kitapci MT. False positive 18F-FDG PET scan in adrenal oncocytoma. Urol Int 2008; 80:444–447. 18587259 10.1159/000132706

[26] Kim SJ Lee SW Pak K Kim I-J Kim K. Diagnostic accuracy of 18F-FDG PET or PET/CT for the characterization of adrenal masses: a systematic review and meta-analysis. Br J Radiol 2018; 91:20170520. 29327944 10.1259/bjr.20170520 PMC6223272

[27] Schaafsma M Berends AMA Links TP Brouwers AH Kerstens MN. The diagnostic value of 18F-FDG PET/CT scan in characterizing adrenal tumors. J Clin Endocrinol Metab 2023; 108:2435–2445. 36948598 10.1210/clinem/dgad138

[28] Fassnacht M Tsagarakis S Terzolo M Tabarin A Sahdev A Newell-Price J. European Society of Endocrinology clinical practice guidelines on the management of adrenal incidentalomas, in collaboration with the European Network for the Study of Adrenal Tumors. Eur J Endocrinol 2023; 189:G1–G42. 37318239 10.1093/ejendo/lvad066

[29] Akkuş G Güney IB Ok F Evran M Izol V Erdoğan S. Diagnostic efficacy of 18F-FDG PET/CT in patients with adrenal incidentaloma. Endocr Connect. 2019; 8:838–845. 31137014 10.1530/EC-19-0204 PMC6599076

[30] Sundin A Hindié E Avram AM Tabarin A Pacak K Taïeb D. A clinical challenge: endocrine and imaging investigations of adrenal masses. J Nucl Med 2021; 62(Suppl 2):26S–33S. 34230070 10.2967/jnumed.120.246066 PMC12530502

